# Cardiologists' knowledge and implementation of lifestyle counselling for cardiovascular disease prevention: A national survey

**DOI:** 10.6026/9732063002001948

**Published:** 2024-12-31

**Authors:** Keerthika Muniasamy, Neya Kavya Chander, Nikitha Gudapati, Sanjit Pradeep, Elangovan Raman, Saranya R., Niveditha Suresh Babu, Srinidhi K.

**Affiliations:** 1 Department of Surgery, Madras Medical College, Chennai, India; 2 Institute of Internal Medicine, Madras Medical College & Rajiv Gandhi Government General Hospital, Chennai, India; 3 Department of Internal Medicine, Rajiv Gandhi Government General Hospital, Chennai, Tamilnadu, India; 4 Department of Internal Medicine, Sri Ramachandra Institute of Higher Education and Research, Chennai, India; 5 Institute of Internal Medicine, Madras Medical College & Rajiv Gandhi Government General Hospital, Chennai, India; 6 Department of Community Medicine, Madha Medical College and Research Institute, Chennai, Tamilnadu, India; 7 Institute of Internal Medicine, Madras Medical College, Chennai, India

**Keywords:** Cardiovascular disease prevention, lifestyle counselling, cardiologists, knowledge, implementation practices, national survey

## Abstract

Cardiovascular disease remains a leading global cause of morbidity and mortality, with lifestyle modification playing a critical role in prevention. This nationwide cross-sectional survey of 100 cardiologists assessed attitudes, practices and knowledge regarding lifestyle counseling for CVD prevention. While most cardiologists recognized the importance of lifestyle counseling, gaps were evident in its consistent implementation, particularly in areas like dietary guidance and physical activity promotion. Key barriers included time and resource constraints and concerns about patient compliance. These findings highlight the need for targeted interventions and training to enhance the integration of lifestyle counseling into clinical practice, fostering a more comprehensive approach to CVD prevention.

## Background:

Cardiovascular disease (CVD) is the most significant cause of death worldwide, accounting for an estimated 17.9 million deaths yearly, or 32% of all deaths globally [1]. The major modifiable risk factors in CVD are hypertension, dyslipidemia, smoking, a sedentary lifestyle and poor dietary habits; so, prevention is the cornerstone in the management of the disease [2]. On the other hand, evidence shows that lifestyle interventions on the basis of diet, physical activity and smoking cessation may significantly reduce CVD incidence and improve patient outcomes [3]. Furthermore, the integration of lifestyle counselling into routine clinical practice remains inconsistent, which arises from both cardiologists' differences in their approach to the lifestyle modification of their patients and the frequency of their provision of counselling [4] . Cardiologists, being the specialists who manage and treat patients with CVD, are therefore best suited to provide lifestyle advice to the patients. There have been several recommendations from major health bodies, including the American Heart Association and the European Society of Cardiology, that lifestyle counselling be considered as first-line intervention in reducing CVD risk and improving health outcomes [5]. However, studies reveal that despite awareness and recognition of lifestyle counselling by most cardiologists, often more constraints such as limited consultation time, insufficient training in behavioural counselling and insufficient resources prevent the routine implementation [6]. Moreover, the patient factors are usually cited as lack of perceived compliance and low motivation, which hinder cardiologists from actually persuading the patients to carry out lifestyle changes [7]. Recently, it has been identified that cardiologists' awareness of particular lifestyle advice, like physical activity or diet, is what influences their involvement in conducting those lifestyle advice [8]. Knowledge is incomplete practice, though. Most cardiologists reportedly discuss lifestyle changes only occasionally [9]. Among the probable causes of this gap are that a preventive care aspect of medical education is not to the best of traditionally sharpened focus and instead more attention is given to pharmacological and procedural treatments in cardiology practice [10]. Therefore, it is of interest to assess the level of knowledge, attitudes and current practices of cardiologists regarding lifestyle counselling for CVD prevention on a national scale.

## Methodology:

This study incorporated a cross-sectional survey-based design to assess the knowledge and practice of cardiologists pertaining to lifestyle counselling in the prevention of CVD. A structured questionnaire based on contribution from a literature review and experts was developed to assess knowledge regarding lifestyle counselling principles, current practice, perceived barriers and attitudes toward its role in preventing CVD among the cardiology clinicians.

## The questionnaire had three sections:

Demographic and practice characteristics, knowledge of lifestyle counselling guidelines and frequency of implementation. Questions on demographics were asked concerning age, years of experience, practice setting and region-for example, whether the respondents' practices were situated in an undisclosed setting, within a hospital, or otherwise. Questions about knowledge of specific lifestyle recommendations-specifically, diet; physical activity and smoking cessation recommendations-and how often they applied were put in both the knowledge and implementation sections. Respondents utilized a Likert scale on implementation items running from never to always for all implementation items and from strongly disagree to strongly agree for knowledge and attitude items.

There were 100 practicing cardiologists participating in the multiple regions across the country. They are intended to account for different years of experience and different settings of practice. The respondents needed to have over one year of clinical experience in cardiology in order to be included. The online-based survey accessed only by the secure web-based portal in order to ensure the anonymous use. Reminder emails every other two weeks during the two months total duration of the data collection. Descriptive statistics included the computing of means and standard deviations obtained from the demographic and the survey responses. The tests that were involved were T-tests and ANOVA to establish differences that may exist in the knowledge and implementations of various demographic groups. For example, the result was compared across years of experience and practice setting and the results were computed at a statistical significance level of p < 0.05. The institutional ethics committee approved the study, with informed consent obtained from all the participants. The study ensured participants' privacy by maintaining anonymity and confidentiality as achieved through voluntary participation according to ethical guidelines set.

## Questionnaire:

The survey tool was designed to include sections such as demographic information, awareness of lifestyle guidelines, current counselling practices, perceived barriers and attitudes toward lifestyle modification. It also included open-ended questions that would elicit specific difficulties cardiologists face while delivering lifestyle counselling to further elaborate on some of these challenges.

## Data collection and analysis:

The survey made use of a mixed-mode approach to allow the options of online and paper-based distribution according to the preference of the participants. Responses to the quantitative questions underwent descriptive statistics while those arising from open-ended questions were thematically coded for common themes. The statistical analysis was performed using SPSS version 26 with p < 0.05 as the levels of significance. The same open-ended responses were utilized qualitatively to examine them while adding more context and depth to the findings of the quantitative analysis.

Table 1 below shows the Summary of the Questionnaire Structure: The questionnaire covered demographics, awareness of lifestyle guidelines, counselling practices, perceived barriers and attitudes toward lifestyle modification, with open-ended questions to explore specific challenges faced in delivering lifestyle counselling.

## Results:

Table 2 presents demographic information, including age, years of experience and practice setting, reflecting a broad cross-section of the cardiologist population. Table 3 details cardiologists' familiarity with established lifestyle counselling guidelines, indicating varied awareness levels. Table 4 highlights how frequently cardiologists counsel patients on lifestyle modifications, showing a gap between awareness and implementation. Table 5 outlines the specific lifestyle areas cardiologists' address during consultations, with a primary focus on exercise and diet. Table 6 presents perceived barriers to lifestyle counselling, with time constraints and patient non-compliance being the most commonly reported. Table 7 reflects cardiologists' attitudes toward the importance of lifestyle counselling in CVD prevention, with a strong consensus on its value. Table 8 shows cardiologists' interest in additional training on lifestyle counselling, indicating a majority are open to further education. Table 9 presents data on resource availability for lifestyle counselling, revealing limited access to specialized materials or support staff. Table 10 outlines cardiologists' perceptions of the effectiveness of lifestyle counselling in improving patient outcomes, with a majority rating it as highly effective. Table 11 summarizes common themes from open-ended responses, with cardiologists noting specific challenges and suggestions for improving lifestyle counselling.

## Discussion:

This paper sheds light on the knowledge, attitudes and practices of cardiologists regarding lifestyle counselling for the prevention of CVD [11]. Findings reveal that even though most cardiologists concur on the critical role of lifestyle interventions in the management of CVD risk, important gaps are still noted regarding the consistent application of such interventions during clinical encounters [12]. Our findings show that most cardiologists have an overview of lifestyle counselling principles, above all the fact that all of these practices of eating habits alteration increased or enhanced exercise levels, quitting smoking may result in lower CVD progression. Of course, all those earlier reviews confirm the point that all cardiologists acknowledged well that, indeed lifestyle intervention works effectively preventing CVD advancement [13]. However, despite that awareness, many cardiologists do not receive full training to prepare them to counsel behaviour adequately and may have something to do with the heterogeneity of lifestyle guidance application in clinical practice [14]. The result calls for more inclusion of behavioural and preventive counseling education in cardiology training programs since better-prepared clinicians are likely to be self-assured on the discussion on lifestyle change with the patients [15]. While cardiologists in general appreciate the merits of lifestyle counselling, our study demonstrates that actual practice is not consistent with such an understanding [16]. The major reasons for non-solicitation most often reported were a lack of time during consultations. Indeed, our findings fit within previous literature evidence indicating time constraints are a significant interference in practicing full-length lifestyle counselling [17]. In clinical practice settings where time is limited, cardiologists tend to focus more often on drug therapies and procedural interventions than on lifestyle counselling because they perceive the latter would take much time [18]. Accessible resources for referral networks also remain inaccessible to cardiologists to help the patient adopt sustainable lifestyle changes [19]. This barrier can also be overcome by developing pathways for multidisciplinary referrals as support staff trained in such lifestyle interventions can be combined [20]. The very reason for this lack of implementation of lifestyle counselling is also due to the perceived lack of adherence in patients. Many cardiologists admit that patients' poor compliance with recommendations to implement lifestyle advice can discourage them from engaging much in such conversations [21]. Motivation and readiness for change of a patient will determine the lifestyle counselling's success and according to research, there are structures of support which have their origin in motivational interviewing: it supports adherence and helps with behaviour change in a long-lasting mode [22]. Incorporating motivational interviewing as part of the cardiologist's practice may lead to better uptake of lifestyle recommendations from patients, which in turn reduces CVD risk [23]. In general, the study findings indicate overreliance on pharmacological and procedural interventions in cardiology and thereby may contribute to a lower priority being placed on advice and counselling of lifestyle. While medications and interventions are part of the management of CVD, more recent research supports the rationale of lifestyle changes as the mainstay for reducing cardiovascular events and providing better long-term health outcomes [24]. A multidisciplinary approach of lifestyle counselling in combination with pharmacological interventions may be beneficial to cardiologists looking to provide patients with effective, long-term care options [25]. Besides, asking patients to try lifestyle adjustments as supplements as opposed to alternatives to medications would give them the perception that non-pharmacologic interventions are part of a complete health plan [26]. Thus, gaps in the lifestyle counselling practices, as identified in this work, offer an indication that changes in focused policies would advance cardiology towards becoming more preventive services-oriented medicine. Implementing structured modules of lifestyle counselling in cardiologists' training and professional development on behavioural counselling can improve the strengths of cardiologists in preventive care delivery [27]. Similar institutional policies that provide additional time for education of patients within the standard times of consultation can also enable cardiologists to manage lifestyle factors more effectively [28]. The support of lifestyle modifications through the use of digital health tools, such as m-apps that aid in the tracking of diet and exercise, can contribute to an intensification of counselling efforts, as patients might monitor their own progress and report it back to their healthcare provider [29]. It is indicated that digital interventions can be an appropriate supplement to traditional lifestyle counselling, which enables cardiologists to monitor the patients' progress better and provide additional support after returning from clinics [30]. Although this study provides an overall view regarding the knowledge and practices of cardiologists, it cannot be free of limitations. Generalization towards all cardiologists is highly restricted due to the strength of the sample size, which is sufficient to get general insight. Further validation of these results could be achieved through future research by larger, multi-centre samples and discussion of variations in lifestyle counselling practice between different sites of care and regions. Longitudinal research can be done to determine the long-term effect of standardized lifestyle counselling training on cardiologists' actual practice over time and investigation on patient outcomes following counselling interventions. The participants in this study recognised their own struggle with lifestyle modification and this was associated with higher CVD counselling practice rates as compared to those who had optimal lifestyle practices [31]. The current review attempts to summarize recent scientific literature related to how lifestyle habits and practices may be employed to lower the risk of CVD and frames this literature as "lifestyle medicine [32]". In addition, the AHA also recommends that patients be screened for depression, given its high incidence in cardiac populations [33].

## Conclusion:

The study highlights that while cardiologists recognize the benefits of lifestyle counseling for CVD prevention, they face challenges such as limited time, low patient compliance and inadequate resources. There is strong interest in further training to enhance the integration of lifestyle interventions into practice. Strengthening institutional support and fostering interdisciplinary partnerships could address these barriers, improving preventive strategies and long-term outcomes for at-risk patients.

## Figures and Tables

**Table 1:** Summary of the questionnaire

**Section**	**Focus Area**	**Details Included**
Demographics	Background information	Age, years in practice, practice setting
Knowledge of Guidelines	Familiarity with CVD prevention guidelines	Awareness of lifestyle counselling guidelines for CVD prevention
Current Counselling Practices	Frequency of lifestyle counselling	How often do you counsel patients on diet, exercise and smoking cessation?
Barriers to Counselling	Potential challenges with implementation	What barriers do you encounter in providing lifestyle counselling?
Attitudes Toward Lifestyle Counselling	Importance of lifestyle counselling	How important do you consider lifestyle counselling in CVD prevention?
Training and Education	Interest in further training	Would you be interested in additional training on lifestyle counselling?
Open-Ended Responses	Additional insights	Please share any specific challenges or recommendations for improving lifestyle counselling in cardiology.

**Table 2:** Demographic characteristics of cardiologists

**Variable**	**Percentage (%)**
Age 25-35	20.5
Age 36-45	42
Age 46-55	27
Age 56+	10.5
< 5 Years' Experience	15
5-15 Years' Experience	47.5
> 15 Years' Experience	37.5
Hospital Practice	60
Private Practice	30
Academic Institution	10

**Table 3:** Familiarity with lifestyle counselling guidelines

**Familiarity with Guidelines**	**Percentage (%)**
Very Familiar	40
Somewhat Familiar	50
Not Familiar	10

**Table 4:** Frequency of lifestyle counselling

**Frequency of Counselling**	**Percentage (%)**
Always	35
Often	45
Occasionally	15
Rarely	5

**Table 5:** Types of lifestyle counselling provided

**Lifestyle Area**	**Percentage (%)**
Exercise	80
Diet	75
Smoking Cessation	60
Stress Management	45

**Table 6:** Barriers to lifestyle counselling

**Barrier**	**Percentage (%)**
Limited Time	65
Patient Non-Compliance	50
Lack of Resources	40
Insufficient Training	35

**Table 7:** Attitudes toward lifestyle counselling

**Attitude**	**Percentage (%)**
Very Important	70
Important	25
Neutral	5

**Table 8:** Interest in additional training

**Interest in Training**	**Percentage (%)**
Very Interested	55
Somewhat Interested	30
Not Interested	15

**Table 9:** Availability of resources for counselling

**Resource Availability**	**Percentage (%)**
Adequate	30
Limited	50
Not Available	20

**Table 10:** Perceived effectiveness of counselling

**Perceived Effectiveness**	**Percentage (%)**
Highly Effective	60
Moderately Effective	30
Minimally Effective	10

**Table 11:** Open-ended responses

**Theme**	**Percentage of Responses (%)**
Need for Better Resources	45
Desire for Additional Training	30
Patient Compliance Issues	25
